# Minicircle-oriP-IFNγ: A Novel Targeted Gene Therapeutic System for EBV Positive Human Nasopharyngeal Carcinoma

**DOI:** 10.1371/journal.pone.0019407

**Published:** 2011-05-05

**Authors:** Yufang Zuo, Jiangxue Wu, Zumin Xu, Shiping Yang, Haijiao Yan, Li Tan, Xiangqi Meng, Xiaofang Ying, Ranyi Liu, Tiebang Kang, Wenlin Huang

**Affiliations:** 1 State Key Laboratory of Oncology in South China, Cancer Center, Sun Yat-Sen University, Guangzhou, People's Republic of China; 2 Institute of Microbiology, Chinese Academy of Science, Beijing, People's Republic of China; 3 Department of Radiation Oncology, Cancer Center, Sun Yat-Sen University, Guangzhou, People's Republic of China; Karolinska Institutet, Sweden

## Abstract

**Background:**

Nonviral vectors are attractively used for gene therapy owing to their distinctive advantages. Our previous study has demonstrated that transfer of human *IFNγ* gene into nasopharyngeal carcinoma (NPC) by using a novel nonviral vector, minicircle (mc), under the control of cytomegalovirus (CMV) promoter was effective to inhibit tumor growth. However, therapies based on CMV promoter cannot express the targeted genes in cancer tissues. Previous studies indicated that the development of human NPC was closely associated with Epstein-Barr virus (EBV) and demonstrated the transcriptional enhancer function of oriP when bound by EBV protein. Therefore, the present study is to explore the targeted gene expression and the anti-tumor effect of a novel tumor-specific gene therapeutic system (mc-oriP-*IFNγ*) in which the transgene expression was under the transcriptional regulation of oriP promoter.

**Methodology/Principal Findings:**

Dual-luciferase reporter assay and ELISA were used to assess the expression of luciferase and IFNγ. WST assay was used to assess the cell proliferation. RT-PCR was used to detect the mRNA level of EBNA1. RNAi was used to knockdown the expression of EBNA1. NPC xenograft models in nude mice were used to investigate the targeted antitumor efficacy of mc-oriP-*IFNγ*. Immunohistochemistry was used to detect the expression and the activity of the IFNγ in tumor sections. Our results demonstrated that mc-oriP vectors mediated comparable gene expression and anti-proliferative effect in the EBV-positive NPC cell line C666-1 compared to mc-CMV vectors. Furthermore, mc-oriP vectors exhibited much lower killing effects on EBV-negative cell lines compared to mc-CMV vectors. The targeted expression of mc-oriP vectors was inhibited by EBNA1-siRNA in C666-1. This selective expression was corroborated in EBV-positive and -negative tumor models.

**Conclusions/Significance:**

This study demonstrates the feasibility of mc-oriP-*IFNγ* as a safe and highly effective targeted gene therapeutic system for the treatment of EBV positive NPC.

## Introduction

The goal of cancer treatment is to selectively eliminate malignant cells while leaving normal tissues intact [1]. Therefore, targeted strategies are needed to be implemented for future therapies to ensure efficient activity at the site of patients' primary tumors or metastases without causing intolerable side effects. To this end, cellular mechanisms of gene regulation have been successfully exploited to direct therapeutic gene expression into cancer cells [2], [3], [4]. Transcriptional targeting is feasible because the tissue- or cancer-specific promoter can be activated in the targeted cancer cells in the presence of proper subset of activators while remaining silent in the non-targeted cells [5].

Nasopharyngeal carcinoma (NPC) is prevalent in South China, North Africa, and among Alaskan Eskimos. A unique feature of NPC is that nearly 100% of anaplastic or poorly differentiated nasopharyngeal carcinomas contain Epstein-Barr virus genomes and express EBV proteins [6], which are expressed exclusively in the malignant tissues but not in the surrounding normal tissues. This difference provides an exploitable opportunity for tumor-specific targeting. Initial genetic dissections of EBV identified one viral protein, Epstein-Barr Nuclear Antigen 1 (EBNA1), and one region of the viral genome, termed latent origin of plasmid replication (oriP), as being necessary and sufficient for replication of the viral plasmid. Previous studies have determined that EBNA1 is essential for regulating the transcription of the transforming genes of EBV [7], [8], [9]. Additionally, EBNA-1, the only viral protein required for the replication of EBV in latently-infected cells, is found in all EBV-associated malignancies [10]. The oriP is composed of two separable *cis* elements, the Family of Repeats (FR) and Dyad Symmetry element (DS) [11]. The FR element consists of 20 tandem 30-bp repeats and acts as a transcriptional enhancer for heterologous promoters when it is bound by EBNA1. Based on these features, the oriP-CMV promoter has been exploited for targeted gene therapy in EBV-positive NPC [12].

Our laboratory has investigated the potential effect of minicircle-mediated *IFNγ* gene therapy in human nasopharyngeal carcinoma. Our data indicated that *IFNγ* gene transfer produced an antiproliferative effect on NPC cells *in vitro* and a profound antitumor effect *in vivo* [13], [14]. We also demonstrated that minicircle-CMV-*IFNγ* was more efficient than corresponding conventional plasmids due to its capability of mediating long-lasting, high level of *IFNγ* gene expression [13]. The CMV promoter, however, is ubiquitously expressed without tumor-targeting activity; thus the application of this promoter is limited due to the potential side effects caused by unwanted expression of a therapeutic gene in normal tissues [15]. Furthermore, the persistence of transgene expression from the minicircle can be achieved in cells with low turnover rates, such as hepatocytes and skeletal muscle cells [16]. These issues are especially critical when the therapeutic gene is delivered systemically, such as by intravenous injection.

To solve these problems, we have developed a novel minicircle (mc) vector in which transgene expression is under the transcriptional regulation of the oriP-CMV promoter (hereinafter to be referred as oriP promoter). The binding of EBNA1 to the FR domain in oriP region activates the transcription of downstream genes. Selective expression of the therapeutic gene is successfully achieved both *in vitro* and *in vivo*, indicating the feasibility of mc-oriP-*IFNγ* as a safe and highly effective gene therapy system for the treatment of NPC. To our knowledge, this is the first report on a non-viral minicircle vector used in targeted gene therapy and is the first time that the oriP promoter has been combined with the minicircle system for NPC-targeted therapy. This strategy lays the foundation for targeted gene therapy of metastatic NPC by intravenous delivery of therapeutic genes.

## Results

### Selective expression of luciferase in EBV-positive C666-1 cells mediated by oriP-vectors

**Figure 1:**
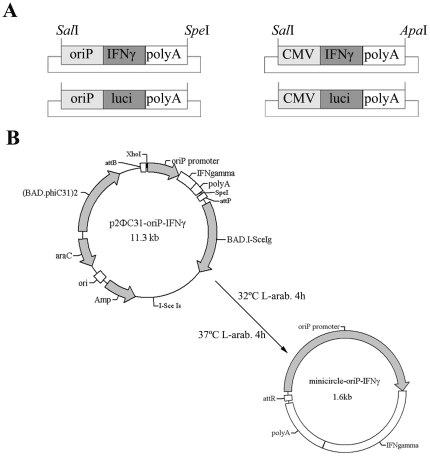
Schematic diagram of the construction of mc-oriP and control vectors and the generation of mc-oriP-IFNγ. (**A**) Diagram of the minicircle vectors carrying the IFNγ gene or firefly luciferase gene under the control of the oriP promoter (left) or CMV promoter (right). (**B**) Flow chart of ФC31 integrase-mediated intramolecular recombination of p2ФC31-oriP-IFNγ. The resulting product is minicircle-oriP-IFNγ. Amp, ampicillin resistance gene; BAD, araBAD promoter; araC, araC repressor; attB, bacterial attachment site; attP, phage attachment site; attR, right hybrid sequence; I-SceIg, I-SceI gene; I-SceIs, I-SceI cutting site; L-arab., L-arabinose.

**Figure 2:**
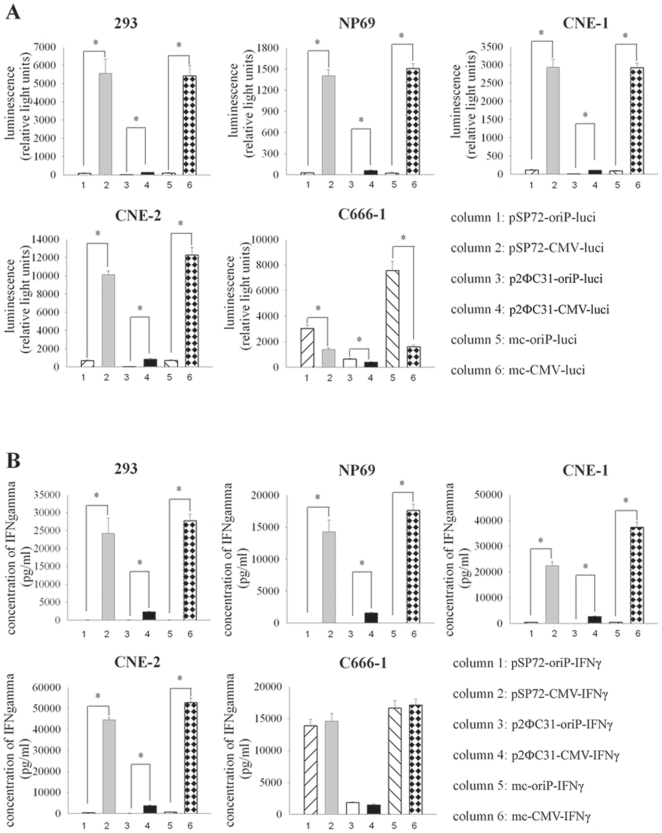
Selective expression of luciferase or IFNγ gene mediated by mc-oriP vector in EBV-positive cells. (**A**) Luciferase activities were assayed using the Dual-Luciferase Reporter Assay System for EBV-negative cell lines (293, NP69, CNE-1 and CNE-2) and the EBV-positive cell line (C666-1). (**B**) The expression level of IFNγ in the supernatant of different cells transfected with plasmids carrying the human IFNγ expression cassette as detected by an ELISA kit. Columns, mean of three independent experiments; bars, SD; *, *p*<0.05, gene expression under the control of oriP promoter compared with CMV promoter.

To determine the transgene expression provided by the novel EBNA1-regulated minicircle vectors in EBV-negative cells (293, NP69, CNE-1 and CNE-2 cells) and the only available EBV-positive NPC cell line (C666-1), the minicircle-luci was compared with its parent plasmid p2ΦC31-luci and the intermediate plasmid from which p2ΦC31-luci was derived (pSP72). All these plasmids contained a luciferase expression cassette driven by an oriP promoter or cytomegalovirus promoter (Figure 1A). Luciferase activity was assessed 48 h (72 h for C666-1 since its low growth rate) after transfection using the dual-luciferase reporter assay system. Luciferase activities were 10–60 fold lower when driven by oriP promoter than by CMV promoter in EBV-negative cells (*p*<0.001; Figure 2A). In contrast, luciferase activity was 2–5 fold higher when driven by oriP promoter than by CMV promoter in the EBV-positive C666-1 cells (*p*<0.01; Figure 2A). Occasionally, low levels of luciferase activity were detected in some EBV-negative cells when driven by the oriP promoter, which suggested the basal expression induced by the minimal CMV IE promoter included in oriP promoter. This observation is consistent with a previous study [12]. The expression levels of the minicircle groups were significantly higher than those of the corresponding parent groups, demonstrating that the minicircle is more efficient in mediating transgene expression *in vitro*. (*p*<0.05; Figure 2A). There was no consistent difference when minicircle groups were compared with pSP72 groups in different cell lines in the short term (48 hrs or 72hrs), which may be due to the size of the pSP72 vector is close to the minicircle vector.

### Selective expression of IFNγ in EBV-positive C666-1 cells mediated by oriP-vectors

To investigate whether mc-oriP-*IFNγ* can mediate efficient expression of the *IFNγ* gene in EBV-positive NPC cells, the minicircle-*IFNγ* was compared with its parent plasmid p2ΦC31-*IFNγ* and the intermediate plasmid from which p2ΦC31-*IFNγ* was derived (pSP72). All of these plasmids contained an IFNγ expression cassette driven by an oriP promoter or CMV promoter (Figure 1A). 293 cells, NP69 cells, and three NPC cell lines were transfected according to the regimen shown in Table 1. The culture supernatant of each treatment was collected to investigate the cumulative production of IFNγ over the indicated time course using ELISA. No IFNγ was found in the culture medium from p2ΦC31-transfected cells (data not shown). The expression level of IFNγ was obviously lower when driven by oriP promoter than by CMV promoter in EBV-negative cells (*p*<0.05; Figure 2B). However, there was no significant difference in IFNγ expression level when driven by the oriP or CMV promoter in EBV-positive C666-1 cells (*p*>0.05; Figure 2B). The expression levels of the minicircle groups were significantly higher than those of the corresponding parent plasmid groups (*p*<0.05; Figure 2B). There was no consistent difference when the minicircle groups were compared with the pSP72 groups in different cell lines.

**Table 1:** Treatment regimens for *in vitro* and *in vivo* transfections.

Treatment regimen used for transfected cells(1 µg DNA +1 µl lipofectamine 2000/well)Group (*in vitro*)	Treatment regimen used for NPC-xenografted mice(15 µg DNA +60 µl lipofectamine 2000*/mouse)Group (*in vivo*)
pSP72-oriP-IFNγ/lucipSP72-CMV-IFNγ/lucip2ΦC31-oriP-IFNγ/lucip2ΦC31-CMV-IFNγ/luciminicircle-oriP-IFNγ/luciminicircle-CMV-IFNγ/lucip2ΦC31	p2ΦC31-oriP-IFNγp2ΦC31-CMV-IFNγminicircle-oriP-IFNγminicircle-CMV-IFNγp2ΦC31

*0.9% NaCl solution was added to adjust the total volume to 100 µl. Each mouse was treated with intratumoral injection of plasmid-liposome complex once a week for 3 weeks.

### Mc-oriP-IFNγ selectively inhibits the growth of EBV-positive C666-1 cells

**Figure 3:**
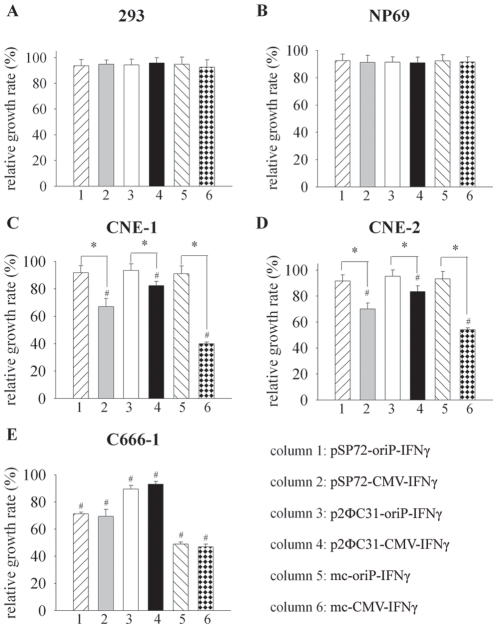
Minicircle-oriP-IFNγ selectively inhibits the growth of EBV-positive C666-1 cells *in vitro*. Cells were treated with minicircle- IFNγ or control plasmids for 48 hours (72 hours for C666-1). Cell viability was determined by WST assay. Data are given as relative growth rates compared with the p2ФC31-treated group. Columns, mean of three independent experiments; bars, SD; #, *p*<0.05, compared with the p2ФC31-treated group; *, *p*<0.05, gene expression under the control of oriP promoter compared with CMV promoter.

The killing effects of mc-oriP-*IFNγ* on NPC cells compared with mc-CMV-*IFNγ* were assessed using the WST assay. The WST assay was performed with Cell Counting Kit-8 (CCK-8) by using Dojindo's highly water-soluble tetrazolium salt WST-8. WST-8 is reduced by dehydrogenases in cells to give a yellow colored product (formazan), which is soluble in the tissue culture medium. The amount of the formazan dye generated by the activity of dehydrogenases in cells is directly proportional to the number of living cells. The detection sensitivity of CCK-8 is higher than other tetrazolium salts such as MTT, XTT, MTS or WST-1 (Cell Counting Kit-8 Technical Manual). Transfection of NPC cells with the mc-CMV-*IFNγ* resulted in significantly reduced cell viability in both EBV-negative cells (CNE-1, CNE-2) and EBV-positive C666-1 cells (*p*<0.05; Figure 3). However, mc-oriP-*IFNγ* caused a significant reduction of C666-1 cell viability but no impact on EBV-negative cells. Treatment of C666-1 cells with mc-oriP-*IFNγ* or mc-CMV-*IFNγ* achieved a similar extent of toxicity (relative growth rate of 48.89±1.7% and 46.89±2.07%, respectively; *p*>0.05), suggesting that the two promoters provided comparable levels of transgene expression. These results are consistent with previous observations reported by Li et al [12]. Furthermore, the minicircle showed more profound effects than the parent plasmid (*p*<0.01). In contrast to NPC cell lines, no growth inhibitory effect was observed in 293 and NP69 cells treated with *IFNγ* gene transfer (*p*>0.05).

### The oriP-based promoter is responsive to EBNA1

**Figure 4:**
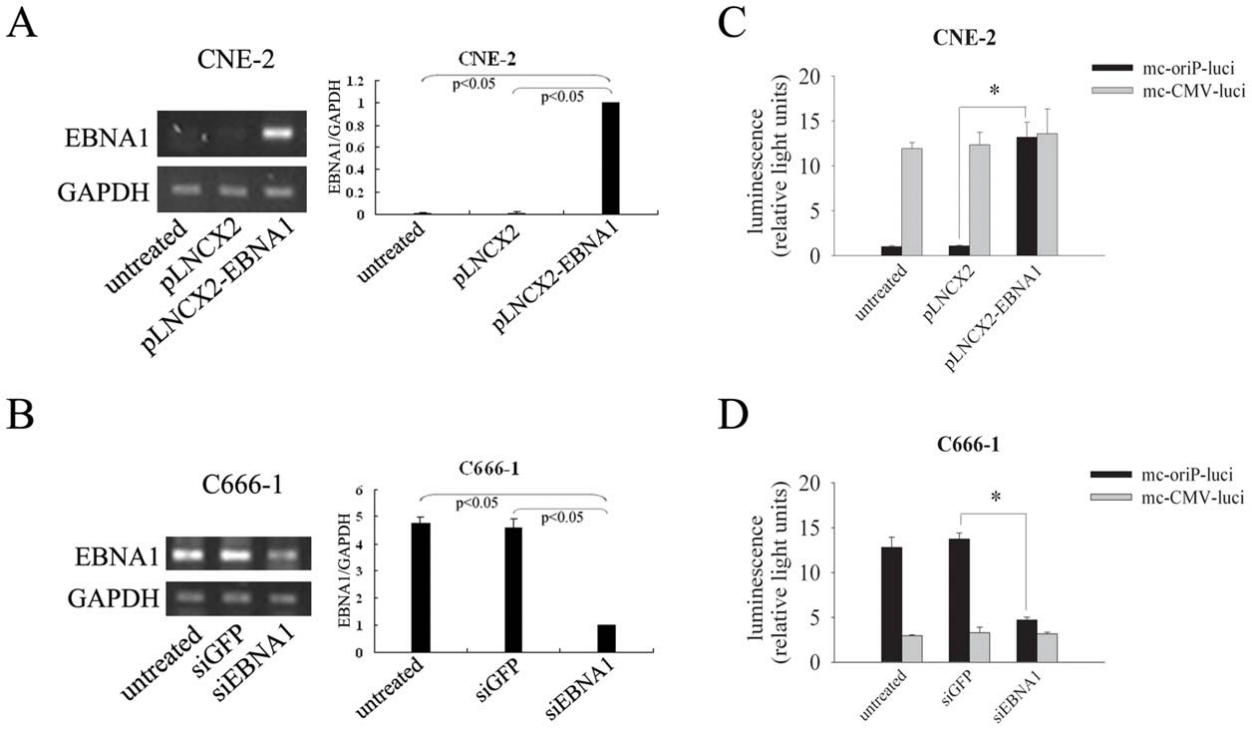
Transcriptional expression and function of EBNA1 in EBV-negative (CNE-2) and -positive (C666-1) NPC cell lines. (**A, B**) Reverse transcription-PCR analysis of EBNA1. A: CNE-2 cells were transiently transfected with plasmid expressing EBNA1 or control plasmid. For quantitative analysis, pLNCX2-EBNA1 group was normalized to 1; B: C666-1 cells were treated with siRNA against GFP (siGFP) or EBNA1 (siEBNA1). For quantitative analysis, siEBNA1 group was normalized to 1. (**C, D**) Luciferase activity was assayed in overexpressing EBNA1 (CNE-2) or silencing EBNA1 expression (C666-1) cell lines followed by transfection with mc-oriP-luci or mc-CMV-luci. Columns, mean of three independent experiments; bars, SD;*, *p*<0.05.

Although endpoint PCR is at best only semi-quantitative, we observed a distinct increase in EBNA1 mRNA level following overexpression (*p*<0.05; Figure 4A) and a clear decrease following siRNA treatment (*p*<0.05; Figure 4B). To further verify whether the targeted gene expression of oriP promoter was regulated by EBNA1 and transient expression of EBNA1 was sufficient to enhance the luciferase activity of the mc-oriP-*luci* treatment group, the following experiments were performed. EBV-negative CNE-2 cells were pre-transfected with an EBNA1 expression plasmid, followed by a second round of transfection with mc-oriP-*luci* 48 hrs later. EBNA1 expression increased the luciferase activity in the mc-oriP-*luci* treatment group (*p*<0.01; Figure 4C) while had no effect on the luciferase activity in the mc-CMV-*luci* treatment group (*p*>0.05; Figure 4C). The EBV-positive C666-1 cells were treated with siRNA to down-regulate EBNA1 expression. EBNA1 silencing reduced the luciferase activity in the mc-oriP-*luci* treatment group (*p*<0.0001; Figure 4D) with no effect on the luciferase activity in the mc-CMV-*luci* treatment group (*p*>0.05; Figure 4D).

### Targeted efficacy of mc-oriP-IFNγ on nasopharyngeal xenograft tumors

**Figure 5:**
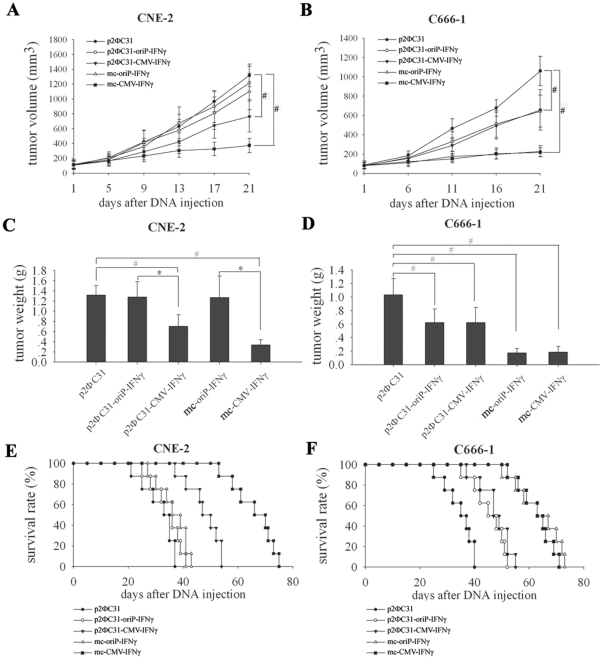
Selective antitumor effects of mc-oriP-IFNγ on the growth of NPC xenografts and survival analysis. (**A, B**) Time-dependent evolution of tumor volume in mice inoculated with the CNE-2 and C666-1 cell lines (n = 6, each group). For CNE-2 cell-xenografted mice, mc-oriP-IFNγ or p2ΦC31-oriP-IFNγ versus p2ΦC31, *p*>0.05; mc-CMV-IFNγ versus p2ΦC31, *p*<0.05 at days 9, 13, 17, and 21; p2ΦC31-CMV-IFNγ versus p2ΦC31, *p*<0.05 at days 13, 17, and 21. For C666-1 cell-xenografted mice, mc-oriP-IFNγ versus p2ΦC31, *p*<0.05 at days 11, 16, and 21; mc-CMV-IFNγ versus p2ΦC31, *p*<0.05 at days 11, 16, and 21; p2ΦC31-oriP-IFNγ versus p2ΦC31, *p*<0.05 at days 16, and 21; p2ΦC31-CMV-IFNγ versus p2ΦC31, *p*<0.05 at days 16 and 21; mc-oriP-IFNγ versus mc-CMV-IFNγ, *p*>0.05. (**C, D**) Specific antitumor effects of mc-oriP-IFNγ on C666-1 cell-xenografted nude mice (n = 6, each group). Mice were sacrificed after three weeks of treatment, and tumors were resected and weighted. Columns, mean of six mice; bars, SD; #, *p*<0.05, compared with p2ФC31-treated group; *, *p*<0.05, gene expression under the control of oriP promoter compared with CMV promoter. (**E, F**) Effect of mc-oriP-IFNγ on survival (n = 8). For CNE-2 cell-xenografted mice, mc-oriP-IFNγ versus p2ΦC31, *p* = 0.066; mc-CMV-IFNγ versus p2ΦC31, *p*<0.0001. For C666-1 cell-xenografted mice, mc-oriP-IFNγ versus p2ΦC31, *p*<0.0001; mc-oriP-IFNγ versus mc-CMV-IFNγ, *p* = 0.368 (Kaplan-Meier).

To determine whether the *in vitro* data of selective expression of the oriP-driven minicircle vector can be corroborated in a more complex *in vivo* model, CNE-2 (EBV-negative) and C666-1 (EBV-positive) tumors were treated with either mc-oriP-*IFNγ* or mc-CMV-*IFNγ*. Each mouse was treated with intratumoral injection of 100 µl plasmid-liposome complex containing 15 µg DNA and 60 µl lipofectamine 2000 once a week for 3 weeks (as shown in Table 1). The time-dependent evolution of tumor volume in mice inoculated with CNE-2 and C666-1 cells (Figure 5A and B) indicated that treatment with mc-oriP-*IFNγ* had minimal inhibitory effect on EBV-negative CNE-2 tumors compared with the control p2ΦC31 group (Figure 5A). In contrast, the tumor size of EBV-positive C666-1 xenografts treated with mc-oriP-*IFNγ* was significantly decreased compared with control groups (*p*<0.05; Figure 5B). Furthermore, mc-oriP-*IFNγ* treatment had similar effect on EBV-positive C666-1 tumors compared to mc-CMV-*IFNγ* treatment (*p*>0.05).

The inhibition rate of the treated group was determined according to tumor weight (Figure 5C and D), and the growth of tumors after mc-oriP-*IFNγ* or mc-CMV-*IFNγ* treatment was significantly slower than those of the control groups (*p*<0.05). In the CNE-2 cell-xenografted models, the inhibition rates of p2ΦC31-CMV-*IFNγ* and mc-CMV-*IFNγ* groups were 46.57% and 74.6%, respectively (Figure 5C). For the C666-1 cell-xenografted models, the inhibition rates in the p2ΦC31-oriP-*IFNγ*, p2ΦC31-CMV-*IFNγ*, mc-oriP-*IFNγ* and mc-CMV-*IFNγ* groups were 40.05%, 39.75%, 83.01%, and 82.05%, respectively (Figure 5D). In both models, the minicircle group showed more profound antitumor potential than the parent plasmid-treated group (*p*<0.05; Figure 5C and D).

The long-term outcome of *IFNγ* gene transfer was evaluated by the survival rates of mice, using the protocol outlined in Table 1. There was no additional treatment after three weeks of treatment. For CNE-2 cell-xenografted mice, the median survival of the p2ΦC31, p2ΦC31-oriP-*IFNγ*, p2ΦC31-CMV-*IFNγ*, mc-oriP-*IFNγ* and mc-CMV-*IFNγ* groups was 33±2.828, 36±2.739, 47±2.828, 36±3.536, and 66±6.364 days, respectively (Figure 5E). For C666-1 cell-xenografted mice, the corresponding median survivals were 35±3.536, 45±4.243, 47±3.3, 63±6.364, and 63±4.243 days, respectively (Figure 5F). In both models, the minicircle group had a longer survival duration than the parent plasmid-treated group (*p*<0.05; Figure 5E and F).

### Selective expression in EBV-positive C666-1 tumors mediated by mc-oriP vectors

**Figure 6:**
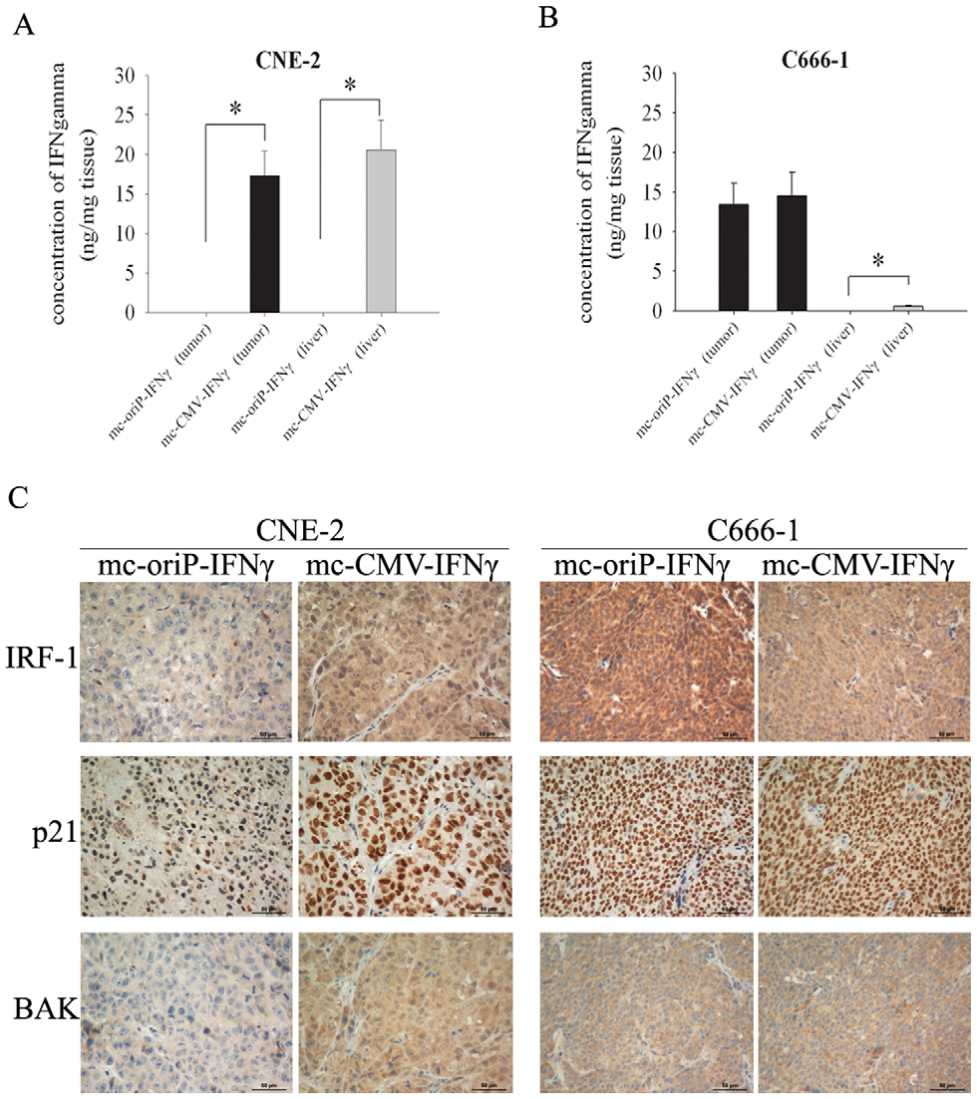
Tissue expression of IFNγ and immunohistochemical analysis. (**A, B**) Expression level of IFNγ in tumor and liver tissue. Results are given in ng/mg of tissue. Columns, mean of three mice; bars, SD. (**C**) Representative immunostaining of IRF-1, p21 and BAK in CNE-2 cell- and C666-1 cell-xenografted tumors treated with mc-oriP-IFNγ or mc-CMV-IFNγ, respectively. Mice were sacrificed after three weeks of treatment, and tumors were resected and frozen for immunohistochemistry assays. IRF-1 staining shows cytoplasmic and nuclear staining, p21 staining shows nuclear staining, and BAK staining shows cytoplasmic staining. In the mc-oriP-IFNγ-treated group, intense IRF-1, p21 and BAK staining were observed only in EBV-positive C666-1 tumors. Tissue sections are shown at ×400 magnification. Scale bar represents 50 µm.

To assay the targeted expression of mc-oriP-*IFNγ in vivo*, IFNγ protein levels were analyzed in tumor and liver using a human IFNγ ELISA kit. Intratumoral expression of mc-oriP-*IFNγ* was detected only in the EBV-positive C666-1 tumor, compared with mc-CMV-*IFNγ* treatment (*p*>0.05; Figure 6A and B). Systemic (liver) expression of mc-oriP-*IFNγ* was not detected in mice bearing either the EBV-negative CNE-2 tumor or the EBV-positive C666-1 tumor, compared with mc-CMV-*IFNγ* treatment (*p*<0.01). However, in mc-CMV-*IFNγ* treatment groups, mice bearing the CNE-2 tumor had much higher expression level of IFNγ in their livers than those bearing the C666-1 tumor (Figure 6A and B).

To further verify the above result of different expressions in liver mediated by the mc-CMV vector, we detected the expression of *luciferase* gene carried by minicircle in liver and tumor tissues through immunohistochemistry. Consistent with the above result, in mc-oriP-*luci* treatment groups, the staining of luciferase was observed only in the EBV-positive C666-1 tumor but neither in the EBV-negative CNE-2 tumor nor in all livers of mice tested. However, in mc-CMV-*luci* treatment groups, the staining of luciferase was observed both in the EBV-positive C666-1 tumor and in the EBV-negative CNE-2 tumor. Liver staining of mc-CMV-*luci* was observed mainly in the mice bearing the EBV-negative CNE-2 tumor (Figure S1). The result is similar to previous report [12].

### Intratumoral injection of mc-oriP-IFNγ results in significant IRF-1, p21 and BAK staining only in the EBV-positive C666-1 tumor

We have demonstrated that IFNγ can effectively activate IRF-1 and lead to the inhibition of cell proliferation and the stimulation of cell apoptosis in NPC (unpublished data). IFNγ-induced G0/G1 arrest and apoptosis has been associated with the induction of p21 and upregulation of Bak, respectively (unpublished data). Thus, immunohistochemistry was used to detect the expression of IRF-1, p21 and BAK. The intratumoral injection of mc-CMV-*IFNγ* resulted in significant IRF-1, p21 and BAK staining in both the EBV-negative CNE-2 and the EBV-positive C666-1 tumors, demonstrating that the expression of IFNγ mediated by the minicircle activated the downstream pathway of IFNγ (Figure 6C). In contrast, injection of mc-oriP-*IFNγ* resulted in obvious IRF-1, p21 and BAK staining only in the EBV-positive C666-1 tumor.

### Minicircle mediates more robust antitumor effect than conventional plasmid

In order to compare the antitumor effects of minicircle and its derived plasmid pSP72 *in vivo*, C666-1 cell-xenografted mice were treated with mc-oriP-*IFNγ* and pSP72-oriP-*IFNγ*. Treatment regimen was conducted with the same protocol as shown in Table 1. Our results indicated that mc-oriP-*IFNγ* could mediate more robust antitumor effect than pSP72-oriP-*IFNγ* at days 16 and 21 (*p*<0.05) (Figure S2).

## Discussion

Minicircle-mediated gene therapeutic techniques shown in previous studies were non-specific, which limited their application due to the potential systemic side effects. We report here for the first time both the non-viral minicircle vector used in targeted gene therapy and the combination of oriP promoter with minicircle system for NPC- targeted therapy. Our findings demonstrate that mc-oriP-*IFNγ* induces antiproliferation of EBV-positive tumor cells, represses tumor growth, and prolongs the mouse life span in a manner as effective as that of mc-CMV-*IFNγ in vitro* and *in vivo*. In addition, mc-oriP-*IFNγ* had minimal or no killing effects on EBV-negative tissues. Therefore, this study indicates the feasibility of mc-oriP-*IFNγ* as a safe and highly effective gene therapy system for NPC treatment.

Nasopharyngeal carcinoma has been shown to be the human tumor showing the most consistent association with EBV [8]. Utilization of molecules uniquely present within a tumor cell to elicit a cytotoxic response is an attractive strategy for the development of new approaches to the targeted therapy of neoplasia. The oriP promoter has been demonstrated to be very powerful in inducing targeted gene expression [12], [17], [18], [19], [20]. In previous studies, the oriP promoter was placed in adenovirus vectors, which achieved very high transduction efficiencies. Although viral-based systems have shown high transfection efficiencies *in vivo*, they have serious disadvantages such as immunogenicity and inflammatory responses [21].

Non-viral gene delivery strategies are usually based on plasmid DNA carrying the gene of interest. Conventional plasmid vectors include a bacterial backbone and a transcription unit. These sequences, however, may cause undesirable effects such as the production of antibodies against bacterial proteins expressed from cryptic upstream eukaryotic expression signals, changes in eukaryotic gene expression caused by antibiotic resistance markers, and immune responses to CpG sequences [22]. Compared to conventional plasmids, minicircle DNAs devoid of plasmid bacterial sequences are superior as non-viral DNA vector for multiple reasons: (*a*) relative safety due to the reduced numbers of inflammatory unmethylated CpG motifs; (*b*) more efficient transgene expression due to its reduced size; and (*c*) more robust and persistent transgene expression [16], [23], [24]. Previous studies have demonstrated that the use of minicircles may offer a promising avenue for safe and efficacious non-viral-based gene therapies [25], [26], [27], [28]. Based on these superior attributes, we developed a recombinant minicircle vector carrying the human *IFNγ* gene that had antiproliferative and antitumor effects against NPC *in vitro* and *in vivo* and also demonstrated that the antiproliferative effects of *IFNγ* gene transfer on NPC cell lines could be attributed to G0-G1 arrest and apoptosis [13].

Considering the need for selective NPC-specific expression, the minicircle vector used in the current study was constructed such that the CMV enhancer promoter was replaced with the oriP-CMV promoter. The new promoter provides significantly higher levels of luciferase reporter gene expression in EBV-positive NPC cells compared with CMV promoter (*p*<0.05; Figure 2A). Although the results of *IFNγ* gene expression are consistent with those of the reporter gene in EBV-negative cells, there was no significant difference between the mc-oriP-*IFNγ*-treated and the mc-CMV-*IFNγ*-treated groups in EBV-positive C666-1 cells, indicating approximately equivalent levels of *IFNγ* gene expression and cytotoxicity between the respective mc-oriP and mc-CMV vectors in the C666-1 cells (*p*>0.05; Figures 2B and 3). One explanation for these observations is that the expression of different genes is variable and consequently, the cell number for *IFNγ* gene expression was decreased after the cytotoxicity caused by IFNγ.

In the present study, the overexpression of the EBNA1 gene in CNE-2 cells resulted in an increase in luciferase activity in the mc-oriP-*luci* treatment group (Figure 4C). Inhibition of EBNA1 expression in C666-1 cells by EBNA1-specific siRNA subsequently reduced the luciferase activity in the mc-oriP-*luci* treatment group but had no effect on the mc-CMV-*luci* treatment group (Figure 4D). We and others have consistently demonstrated that the oriP-based promoter specifically responds to EBNA1 [12], [18], [19], [20]. Therefore, the tumor-specific promoter oriP can be used for gene therapy in EBV-associated diseases, such as Burkitt's lymphoma (BL), Hodgkin's disease (HD), and post-transplant lymphoproliferative disorders (PTLD).

In addition to investigating the selective antiproliferation effects of mc-oriP-*IFNγ in vitro*, the targeted antitumor effects of mc-oriP-*IFNγ* were assessed in NPC-xenografts. The fact that the *in vitro* data could be replicated in *in vivo* tumor models is exciting. Our data show that the antitumor effects and survival rates were similar between the mc-oriP-*IFNγ*-treated and mc-CMV-*IFNγ*-treated groups in the C666-1 tumor model (*p*>0.05; Figure 5). However, the oriP promoter was much less active in the EBV-negative CNE-2 tumor xenografts, resulting in very limited antitumor effect and a reduced survival rate (*p*>0.05; Figure 5). Again, the minicircle was much more efficient than the parent plasmid p2ФC31 and conventional plasmid pSP72 (*p*<0.05; Figure 5 and Figure S2), this may be due to the fact that minicircle DNA does not contain extraneous plasmid backbone sequences that could cause transcriptional repression *in vivo* [23], [29], which is consistent with previous studies [13], [30], [31].

Transgene expression can be detected in the host liver with extensive intratumoral injection of the mc-CMV-*IFNγ* (Figure 6A and B), which raises a concern about potential systemic cytotoxicity. Furthermore, there is a big difference in expression of mc-CMV-*IFNγ* in the livers of mice carrying different tumors. However, the reason for this unexpected result is unclear. A possible explanation is that the tissues of CNE-2 tumors that had higher amount of stroma are looser and softer than those of C666-1 tumors, which in turn may result in more minicircle DNAs in liver probably by the leakage. The underlying mechanism needs further to be elucidated. In addition, transgene expression in the mc-oriP-*IFNγ*-treated groups has not been detected in liver tissues (Figure 6A and B). These results provide support for the mc-oriP-*IFNγ* plasmid as a promising and NPC-specific vector for gene therapy. The minicircle vector is also a versatile tool to carry other tissue- or cancer-specific promoters for the development of promising therapeutics for other diseases.

Orthotopic models of many tumor types have been developed in which metastasis occurs in a similar manner as in patients [32]. Data on orthotopic human NPC models, however, are still limited. In one report investigating this issue, a fluorescent orthotopic NPC metastatic model was established in nude mice using stable and high GFP-expressing NPC cell lines. This model will be useful for understanding the biology of metastatic NPC and for discovering effective therapies for this disease [33]. The next step in developing the oriP-based minicircle system will be to investigate its antimetastatic effect on the orthotopic human NPC model in nude mice. This study will lay the foundation for targeted gene therapy of metastatic NPC by intravenous delivery of a therapeutic gene.

## Materials and Methods

### Cells and culture conditions

The cell lines used in this study were 293 (human embryonic kidney cell line, EBV negative), NP69 (immortalized human nasopharyngeal epithelial cell line, EBV negative), CNE-1 (well-differentiated NPC cell line, EBV negative), CNE-2 (poorly-differentiated NPC cell line, EBV negative), and C666-1 (undifferentiated and the only available EBV positive NPC cell line) [34], [35]. CNE-1, CNE-2 and C666-1 cells were maintained in RPMI 1640 containing 100 units/mL penicillin, 100 µg/mL streptomycin, and 10% fetal bovine serum (Gibco, Paisley, United Kingdom) at 37°C in a 5% CO_2_ humidified atmosphere. NP69 cells were maintained in Keratinocyte-SFM (Gibco, Invitrogen, Cat.10724), and the experiments were conducted when the cells were in an exponential growth phase. C666-1 is a kind gift from Dr. Saiwah Tsao (University of Hong Kong, Hong Kong, PR China). NP69 was kindly provided by Professor Musheng Zeng (State Key Laboratory of Oncology in South China, Cancer Center, Sun Yat-sen University, Guangzhou, PR China). 293, CNE-1, and CNE-2 cell lines were maintained by our lab [13].

### Construction of recombinant parent plasmids

Plasmid p2ФC31 (9.7 kb) was a kind gift from Dr. Zhiying Chen (Stanford University, Stanford, CA) [24]. Plasmid PDC312.oriP.luc (6 kb) carrying the oriP-CMV promoter was provided by Dr. FeiFei Liu (Department of Radiation Oncology, Princess Margaret Hospital University Health Network, Toronto, Ontario, Canada) [12]. pShuttle-*IFNγ* (4.6 kb) carrying the human *IFNγ* expression cassette was constructed by our lab. pSP72 (2462 bp) was obtained from Promega (Madison, WI). pcDNA3.1 (5428 bp) and the *E. coli* strains Top 10 were purchased from Invitrogen.

An 897-bp *Sal*I-*Hind*III fragment containing the EBV oriP-FR region and basal CMV IE promoter from plasmid PDC312•oriP•luc was subcloned into the *Sal*I-*Hind*III sites of the pSP72 plasmid to create pSP72-oriP. The polyA sequence was amplified by PCR from the pcDNA3.1 plasmid and subcloned into the downstream of the oriP promoter in pSP72-oriP plasmid to create pSP72-oriP-polyA. The *IFNγ* gene was amplified by PCR from the pShuttle-*IFNγ* plasmid and subcloned into the pSP72-oriP-polyA plasmid to create intermediate plasmid pSP72-oriP-*IFNγ*. Parent plasmid p2ФC31-oriP-*IFNγ* (11.3 kb) (Figure 1B) was constructed by inserting the 1.6-kb *Sal*I-oriP-IFNγ-polyA-*Spe*I fragment from the above intermediate plasmid pSP72-oriP-*IFNγ* into the *Xho*I-*Spe*I sites of p2ФC31 (*Sal*I and *Xho*I are isocaudamers).

Intermediate plasmid pSP72-oriP-*luci* was constructed by replacing *IFNγ* gene of pSP72-oriP-*IFNγ* with *luciferase* gene obtained from plasmid PDC312-oriP-luc. Then parent plasmid p2ФC31-oriP-*luci* (12.5 kb) was constructed by inserting the 2.8-kb *Sal*I-oriP-luciferase-polyA-*Spe*I fragment from the above intermediate plasmid pSP72-oriP- *luci* into the *Xho*I-*Spe*I sites of p2ФC31.

Intermediate plasmids pSP72-CMV-*IFNγ* and pSP72-CMV-*luci* were constructed by replacing oriP promoter of pSP72-oriP-*IFNγ* and pSP72-oriP-*luci* with CMV promoter amplified by PCR from the pcDNA3.1 plasmid, respectively. Then parent plasmids p2ФC31-CMV-*IFNγ* (11.1 kb) and p2ФC31-CMV-*luci* (12.3 kb) were constructed by inserting the 1.4-kb *Sal*I-CMV-IFNγ-polyA-*Apa*I fragment and 2.6-kb *Sal*I-CMV-luciferase-polyA-*Apa*I fragment from the above intermediate plasmids into the *Xho*I-*Apa*I sites of p2ФC31, respectively. All constructs were confirmed by DNA sequencing (Figure 1).

### Production and purification of minicircles

Minicircle-*IFNγ* and minicircle-*luciferase* were produced according to the methods described by Chen et al. [24] with minor modifications. Briefly, overnight bacterial growth from a single colony of parent plasmid-transformed E. coli Top 10 in Tris-borate medium was centrifuged at 20°C and 4,000 rpm for 20 minutes. The pellet was resuspended 4∶1 (v/v) in fresh Luria-Bertani broth containing 1.5% L-arabinose. The bacteria were incubated at 32°C with constant shaking at 250 rpm for 4 hours. After adding one-half volume of fresh Luria-Bertani broth (pH 8.0) containing 1.0% L-arabinose, the incubation temperature was increased to 37°C, and the incubation was continued for an additional 4 hours. Episomal DNA circles were prepared from bacteria using plasmid purification kits from Qiagen (Chatsworth, CA).

### Quantitative evaluation of mc-oriP-luciferase or mc-CMV-luciferase expression

To evaluate transgene expression from mc-oriP-*luciferase* or mc-CMV-*luciferase* in EBV-negative and -positive cells, luciferase activity was measured using the Dual-Luciferase Reporter Assay System (Promega). Cells were seeded in 24-well culture plates (5×10^4^ cells/well for C666-1, and 3×10^4^ cells/well for other cells). After one doubling, cells were cotransfected with mc-oriP-*luciferase* and pGL4.73 (Promega) simultaneously at a ratio of 50∶1 (Table 1). Cell lysates were analyzed for luciferase activity using the Dual-Luciferase Reporter Assay System and a Luminometer (BERTHOLD Technologies, Centro LB-960) according to the manufacturers' protocols [12].

### Quantitative evaluation of mc-oriP-IFNγ or mc-CMV-IFNγ expression

To evaluate transgene expression from mc-oriP-*IFNγ* or mc-CMV-*IFNγ* in EBV-negative and -positive cells, the concentration of IFNγ in the culture supernatant of transfected cell lines was measured with a human IFNγ ELISA kit (R&D Systems, Minneapolis, MN) according to the manufacturer's protocol. The culture supernatant of transfected cells treated with mc-*IFNγ* for 48 hours (C666-1 for 72 hours since its low growth rate) was collected and frozen (−70°C) for activity analysis [13]. The sensitivity of the kit was up to 16 pg/ml.

### Effect of mc-oriP-IFNγ or mc-CMV-IFNγ on cell viability

To evaluate the effect of mc-oriP-*IFNγ* or mc-CMV-*IFNγ* treatment on viability, the WST assay was used as described previously [13], [36]. WST assay was performed using Cell Counting Kit-8 which was nonradioactive, allowed sensitive colorimetric assays for the determination of the number of viable cells in cell proliferation and cytotoxicity assays. In brief, EBV-negative and -positive cell lines were transfected with mc-oriP-*IFNγ* and corresponding control plasmids (Table 1). After 48 hours (72 hours for C666-1), cell viability was measured with the Cell Counting Kit-8 (Dojindo Molecular Technologies, Inc., Gaithersburg, MD) according to the manufacturer's instructions.

### Transfections and RNA interference

Transfections were conducted according to the manufacturer's instructions with minor modifications. Briefly, to generate CNE-2 cells expressing EBNA1 transiently, 2×10^5^ cells were transfected with 5 µg of the EBNA1 expressing plasmid pLNCX_2_/EBNA1 using 5 µL of lipofectamine 2000 (Invitrogen). For RNA interference experiments, 3×10^5^ C666-1 cells were transfected with the indicated amounts of siRNA against GFP (GCAAGCUGACCCUGAAGUUCAU) or against EBNA1 (GGAGGUUCCAACCCGAAAU) using 5 µL of lipofectamine 2000 [37], [38]. Twenty-four hours later, cells were split for either subsequent transfection or RT-PCR analysis.

### RNA preparation and reverse transcription-PCR

Total RNA was prepared using the Micro-to-Midi Total RNA Purification System (Invitrogen) according to the manufacturer's instructions. RNA was submitted to DNase digestion and 1 µg aliquots were used for reverse transcription with the Reverse Transcription System (Promega). PCR reactions were performed using the following primers: human GAPDH, sense 5′-AGAAGGCTGGGGCTCATTTG-3′ and antisense 5′-AGGGGCCATCCACAGTCTTC-3′; and human EBNA1, sense 5′-AAGGAGGGTGGTTTGGAAAG-3′ and antisense 5′-TGGAATAGCAAGGGCAGTTC-3′. The PCR reaction for EBNA1 was carried out using the following conditions: denaturation—95°C (30 s), annealing—62°C (30 s), and extension—72°C (30 s), with 40 cycles [37]. For GAPDH, the following reaction conditions were used: denaturation—95°C (30 s), annealing—58°C (30 s), and extension—72°C (30 s), with 25 cycles. The sizes of the PCR products were 258 bp for *GAPDH* and 206 bp for *EBNA1*, respectively. ImageJ software was used for quantitative analysis of EBNA1/GAPDH from three independent experiments.

### Antitumor activity of mc-oriP-IFNγ in a subcutaneous NPC tumor model

Care, use, and treatment of all animals in this study were in strict agreement with the institutionally approved protocol according to the USPHS Guide for the care and use of laboratory animals, as well as the guidelines set forth in the Care and Use of Laboratory Animals by the Sun Yat-sen University.

Female BALB/c nude mice (4–6 weeks old) were obtained from Shanghai Slike Experimental Animals Co. Ltd. (Shanghai, China; animal experimental license no. SCXKhu2007–0005). After 1 week of adaptation, the mice were inoculated s.c. in the scapular region with 2×10^6^ CNE-2 cells or 1×10^7^ C666-1 cells to generate tumors for the following experiments. Once the tumor dimension reached approximately 5–8 mm (100 mm^3^), the animals were randomly assigned to groups. Each mouse was treated with intratumoral injection of 100 µl plasmid-liposome complex once a week for 3 weeks, according to the regimen shown in Table 1. For the antitumor experiments, a total of 30 mice were used for either xenograft model (6 mice per group, 5 groups). Tumor volume (V) was measured and calculated according to the following formula: V  =  L×W^2^/2 (L, length; W, width). Tumors were resected at the end point and frozen (−70°C) for analysis. For survival studies, there were eight mice in each group. Five treatment groups were included for either xenograft model. Animals were either found dead or sacrificed when tumors were observed by palpation to approach 10% of the body weight or when individual animals seemed to be stressed by weight loss, ruffled fur, and/or lethargy. All the animal experiments were conducted in accordance with the Guidelines for the Welfare of Animals in Experimental Neoplasia [13].

### IFNγ production by minicircle-IFNγ transfected tumor tissues

Frozen samples were sonicated in 1×TBS (25 mmol/L Tris, 138 mmol/L NaCl, and 3 mmol/L KCl, pH 7.4) and centrifuged at 8,000×g for 1 minute. The resulting supernatants were used for analysis. IFNγ levels were determined using a human IFNγ ELISA kit (R&D Systems) according to the manufacturer's recommendations.

### Antibodies and immunohistochemistry

The following commercial antibodies were used: IRF-1 (Beijing Biosynthesis Biotechnology Co., Ltd, China), p21 and BAK (Boster Biological Technology Ltd, Wuhan, China), Firefly Luciferase (Abcam Inc, UK). Sections of 5 µm from formalin-fixed and paraffin-embedded tumors were deparaffinized in two 5-min washes with xylene and rehydrated through a graded alcohol series to distilled water. The sections were then treated with 0.3% H_2_O_2_ in methanol for 15 min to block endogenous peroxidase activity. Before applying the primary antibody, sections were microwaved for antigen retrieval in 10 mmol/L citrate buffer (pH 6.0) for a total of 25 min, followed by equilibration in phosphate-buffered saline. Sections were treated first at room temperature for 30 minutes with goat serum blocking solution (Boster Biological Technology Ltd, Wuhan, China) in humidity chambers and then incubated with the respective primary antibody for 12–16 h at 4°C. After several washes, the sections were treated with the appropriate secondary antibody (Envison Kit; DAKO) for 30 min. The antigen-antibody complex was visualized by incubation with the DAB Kit (Envison Kit; DAKO). Finally, all sections were counterstained with hematoxylin. All immunostaining was first optimized in single tissue slides. Negative controls were obtained using the method as described above but without incubation with the appropriate primary antibody [39], [40], [41], [42], [43].

### Statistical analysis

All results were evaluated using Student's *t* test with SPSS 11.0 software (SSPS, Inc., Chicago, IL). The survival results were evaluated using Kaplan-Meier curves. *p*<0.05 was considered statistically significant. Representative results from three independent experiments are shown, and the data were presented as mean ± SD.

## Supporting Information

Figure S1 **Immunohistochemical staining of tumor and liver cells expressing luciferase.** CNE-2 or C666-1 s.c. tumors were intratumoral injected with 15 μg of either mc-oriP-*luci* or mc-CMV-*luci*. The mice were sacrificed 72 hours after treatment, and representative images of tumor and liver sections stained for luciferase were obtained. Tissue sections are shown at ×200 magnification. Scale bar represents 50 μm.
(TIF)

Figure S2 **Antitumor effect of mc-oriP-IFNγ compared with conventional plasmid pSP72-oriP-IFNγ.** pSP72-oriP-*IFNγ* versus p2ΦC31, *p* < 0.05 at days 11, 16, and 21; mc-oriP-*IFNγ* versus p2ΦC31, *p* < 0.05 at days 11, 16, and 21; pSP72-oriP-*IFNγ* versus mc-oriP-*IFNγ*, *p* < 0.05 at days 16 and 21. *, *p* < 0.05, pSP72-oriP-*IFNγ*-treated group compared with the mc-oriP-*IFNγ*-treated group.
(TIF)
