# Supplementary figures and images for: Minicircle-oriP-IFNγ: A Novel Targeted Gene Therapeutic System for EBV Positive Human Nasopharyngeal Carcinoma
Source: PLoS One. 2011 May 5;6(5):e19407. doi: 10.1371/journal.pone.0019407 (PMC3088667; doi:10.1371/journal.pone.0019407)

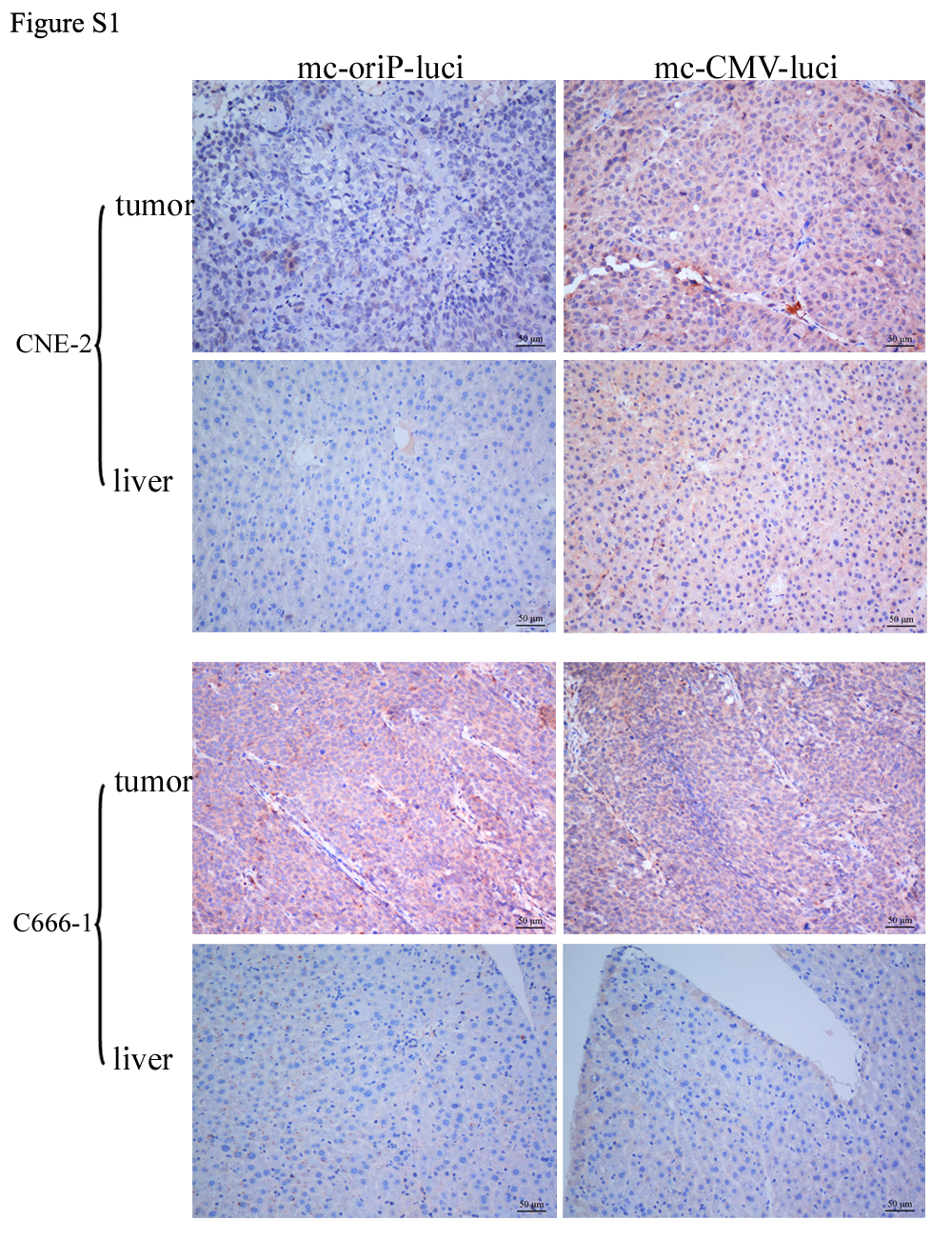

Supplement: Figure S1 — Immunohistochemical staining of tumor and liver cells expressing luciferase. CNE-2 or C666-1 s.c. tumors were intratumoral injected with 15 μg of either mc-oriP-luci or mc-CMV-luci. The mice were sacrificed 72 hours after treatment, and representative images of tumor and liver sections stained for luciferase were obtained. Tissue sections are shown at ×200 magnification. Scale bar represents 50 μm. (TIF) [file pone.0019407.s001.tif]

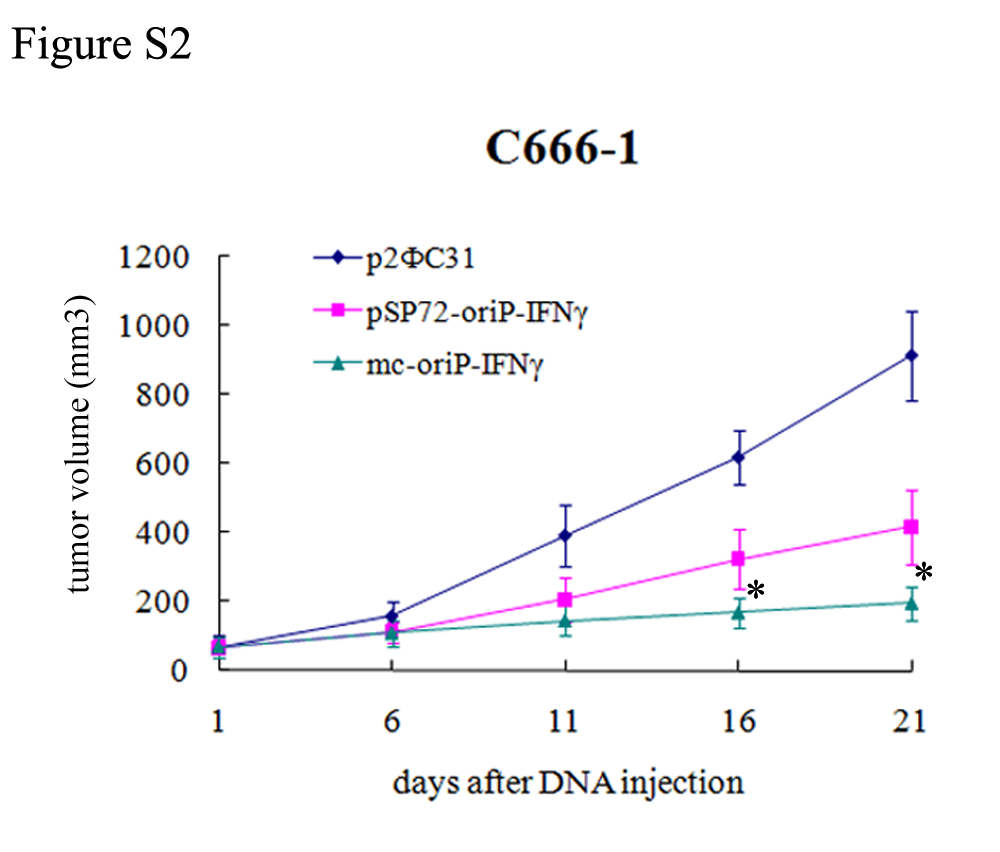

Supplement: Figure S2 — Antitumor effect of mc-oriP-IFNγ compared with conventional plasmid pSP72-oriP-IFNγ. pSP72-oriP-IFNγ versus p2ΦC31, p < 0.05 at days 11, 16, and 21; mc-oriP-IFNγ versus p2ΦC31, p < 0.05 at days 11, 16, and 21; pSP72-oriP-IFNγ versus mc-oriP-IFNγ, p < 0.05 at days 16 and 21. *, p < 0.05, pSP72-oriP-IFNγ-treated group compared with the mc-oriP-IFNγ-treated group. (TIF) [file pone.0019407.s002.tif]
